# The status of citrate in the hydroxyapatite/collagen complex of bone; and Its role in bone formation

**DOI:** 10.7243/2050-1218-3-4

**Published:** 2014

**Authors:** Leslie C. Costello, Meena Chellaiah, Jing Zou, Renty B. Franklin, Mark A. Reynolds

**Affiliations:** 1 Department of Oncology and Diagnostic Sciences, School of Dentistry, University of Maryland, Baltimore, Md. 21201, USA; 2 Department of Periodontics, Dental School, University of Maryland, Baltimore, Md. 21201, USA

**Keywords:** Citrate, calcium, collagen, bone, osteoblasts, hydroxyapatite/collagen complex

## Abstract

**Background:**

It has been known for more than 70 years that citrate is a major component of bone; comprising 1–2% weight of bone, and a concentration that is ~5–25-fold greater than the citrate concentration of most other tissues. This relationship exists in humans and in all vertebrates; which reveals that it is an indispensible and essential structural/functional component of bone. However, its implications relating to the structure and properties of bone, to the process of bone formation and regeneration, to bone disorders, and other issues have remained largely unknown and unaddressed. Recent studies have identified citrate as a structural component of the apatite nanocrystal/collagen complex, which is essential for imparting the bone properties of stability, strength, and resistance to fracture. This raises the issues of the status of citrate, and its source in normal bone formation.

**Methods:**

The present report investigated the association of citrate with the hydroxyapatite (mineral) component and with the collagen component of human cortical bone preparations. The bone preparations were subjected to demineralization procedures to extract the mineral component; followed by extraction of the collagen component in the residual demineralized bone. The extracts were assayed for citrate, calcium, and collagen.

**Results:**

The results reveal, for the first time, the existence of two major pools of citrate in bone. One pool comprising ~65–80% of the total citrate is associated with the hydroxyapatite component; and another pool comprising ~20–35% of the total citrate is tightly bound to the collagen component of the apatite nanocrystal/collagen complex.

**Conclusions:**

Citrate is an indispensible chemical and structural component of the apatite nanocrystal/collagen complex; and is required for manifestation of the biomechanical properties of bone. These results lead to a new concept of bone formation in which citrate incorporation (“citration”) in concert with mineralization must be included in the process of bone formation. Along with this relationship, osteoblast citrate production has recently been identified as the likely source of citrate. It is now evident that the role of citrate in normal bone formation and its implications in bone disorders and defects, and in bone repair and regeneration, now requires renewed attention and support for much needed research.

## Introduction

Dickens in 1941 [1] first reported that bone contained extremely high concentrations of citrate; a relationship that has been confirmed and established by many pursuant reports (reviewed in [2]). Most reports estimate the citrate concentration to be in the range of ~20–100 μmols/gram dry weight. For comparison with soft issues, this would translate to ~5–25 μmols/gram wet-weight; which contrast with soft tissues of <1μmol/gram wet-weight (with some exception such as prostate ~10 μmols/gram). About 90% of the total citrate found in the body resides in bone. Most notably, this high citrate concentration in bone is conserved in all “osteo-vertebrates”; as an evolutionary advancement from “chondro-vertebrates”. This attests to the fact that citrate must have an indispensable important role in the structural and functional properties of normal bone.

The discovery of this citrate relationship over seventy years ago initiated intense research by early investigators into the source and role of citrate in the structure of bone, its implications in bone formation and resorption, and other critical issues. However, these issues remained largely unresolved and/or highly speculative; due mainly to the absence of necessary research methodology and technology. Moreover, beginning ~1975, interest and research into these relationships of citrate as a major component of bone declined. Consequently, contemporary clinicians and biomedical investigators have largely ignored, or are unaware of the existence of citrate in bone; to the extent that it is not even described in recent textbooks and reviews of bone physiology and pathology [3,4].

However, recent NMR/x-ray diffraction studies of bone by Hu et al., [4–6] and by Davies et al., [7] have identified that citrate is a bound component of the apatite nanocomposite-collagen complex; and is essential for imparting the important biomechanical properties of bone such as its stability, strength, and resistance to fracture. This relationship reveals an indispensable role of citrate that has critical implications in virtually every aspect of bone; such as skeletal growth and development, injury and bone disorders, bone repair and regeneration, and more. It is especially notable that, despite the growing application of bone implants and regenerative medicine for osteoinductive bone formation, there exists no reported studies of the status of citrate in the bone product as compared to the host normal bone formation. This is especially relevant since osteogenesis that results in a bone product that does incorporate citrate as exists in normal bone, will not exhibit the manifestation of the important biomechanical properties of normal bone [4–6].

In this report, we focused on the status of citrate in bone in relation to the apatite/collagen complex. The results reveal (for the first time as best that we can determine) the existence of two important major pools of citrate: apatite-associated citrate; which, in combination with calcium, is readily extracted by demineralization of bone; and following demineralization, another pool of citrate that is strongly bound to the collagen complex. Thus, a new understanding is evolving of the citrate relationship in bone; which has important implications in normal bone formation, in bone disorders and bone defects, and in osteoinductive regenerative medicine.

## Methods

For this study, we believed it to be important to establish the status of zinc in human bone; rather than from animal bone studies to be translated to human bone. To achieve this we elected to employ bone preparations that we obtained from LifeNet Health, Inc (hereafter referred to as LifeNet). The studies were conducted with their GC-mineralized cortical particulate (250–300 micron) preparations. The bone preparations are obtained from deceased donor material. The donors have been screened and found to be medically suitable for use as a bone donor. The cortical bone is sourced from long bones (femur, tibia, fibula or humerus). The cortical bone is milled into chunks of approximately 1 cm then ground to the final 250–1000 micron range using a Tekmar grinder (the material is kept cool during processing). Final sizing is accomplished using USP sieves. All work is preformed aseptically in a clean room, and processed using the LifeNet proprietary Allowash XG technology (described at (http://www.accesslifenethealth.org/innovation/allowash_xg) with a final low dose of gamma irradiation to allow for a sterility claim. In summary, the process “*removes greater than 99% of bone marrow and blood elements from the internal bone matrix....and renders allograft bio-implants sterile without compromising their biomechanical or biochemical properties.*” In addition, our results below demonstrate that the mineralized cortical bone preparations exhibited citrate and calcium levels within the expected range of reported bone concentrations. Therefore, we are confident that these bone preparations are highly appropriate for the determination of the status of citrate in human bone; and likely better than we could achieve by laboratory preparations of human bone samples.

The mineralized cortical bone particles were subjected to various extraction procedures in accordance with the aim of the experiments. Therefore, the specific extraction procedures are described below, along with the description of the experiment and the results obtained.

The bone extracts were assayed for citrate by the acetic anhydride/pyridine method [9]. Calcium was assayed with the calcium colorimetric assay kit (BioVision, Inc). Collagen was determined by the Sirius Red collagen reagent kit as described by the vendor (Chondrex, Inc). Proteins in the extracts were separated by 8% SDS-polyacrylamide gel electrophoresis and stained with Coomassie blue; and collagen was identified by Western blot analysis with collagen type 1 antibody (Sigma-Aldrich).

The experiments were conducted in duplicate or triplicate to establish the consistency of the results, which are represented in the following Results section.

## Results

Since reported citrate concentrations of normal bone vary considerably over a range of ~20–100 μmols/gram, we determined the concentration of total citrate in bone particle preparations that were to be employed in this study. For this, we “solubilized” the bone particles in 1M HCl with stirring at 65C for 1 hour; followed by homogenization in a motor-driven Dounce homogenizer; followed by sonication until the presence of bone particles was no longer visible. This extract was assayed for citrate to represent the total concentration of citrate in the mineralized bone. The preparations generally exhibited total citrate concentrations from ~60–100 μmols/gram (~1.2–2.0% gram weight) which is in the expected range of citrate values as reported by others [2]. Consequently, we established that this source of human bone was suitable for the following studies of the status of citrate in bone.

The major aim of this study was to determine the relative pool of the total citrate in bone that might be associated with the hydroxyapatite component and with the collagen component, both of which comprise the hydroxyapatite nanocomposite/collagen complex of bone. We focused on calcium because of its relationship for the binding of citrate in the mineral apatite component and in the apatite-collagen complex component (discussed below). To achieve this, we employed procedures to obtain serial extractions of the mineralized human cortical bone preparations designed to obtain the apatite mineral fraction (demineralization), followed by extraction of the protein/collagen component of the demineralized bone. This required a demineralization procedure that would result in efficient extraction of the mineral component without accompanying extraction of the collagen component.

### Citrate composition of cortical bone extracted with 0.5 M HCl

**Figure 1:**
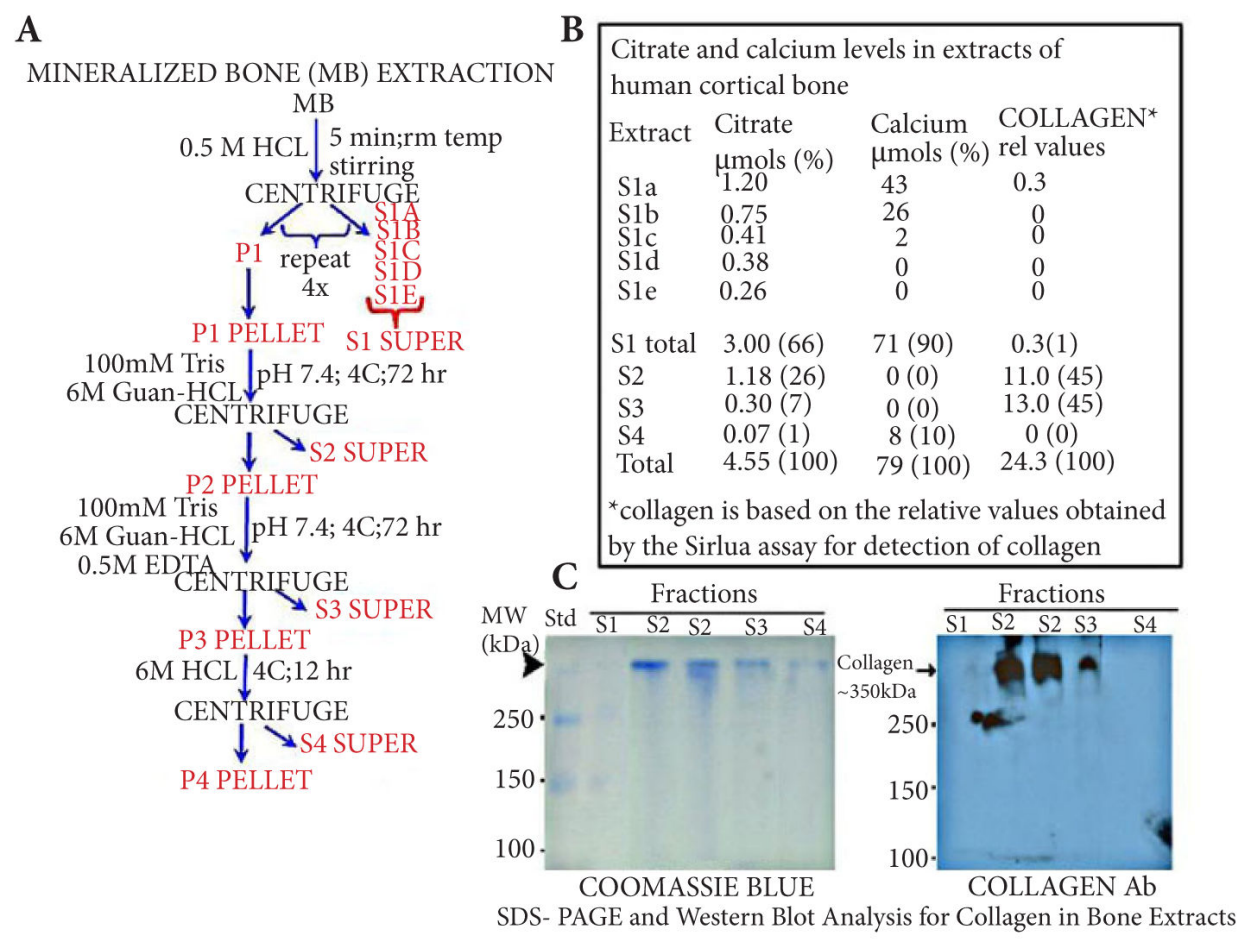
The relative citrate and calcium composition in the mineral and protein extracts of human cortical bone preparation (**A**) The demineralization/protein extraction procedure. (**B**) Citrate and calcium levels of the bone extracts (%=% of total). (**C**) Determination of protein (Coomassie Blue stain) and collagen (Western blot) composition of the bone extracts.

The extraction procedure employed in the following experiment is represented in Figure 1. The mineral component was obtained by employing a relatively mild HCl extraction; so as to eliminate or minimize any accompanying extraction of collagen from the apatite-collagen complex. The procedure was based on a demineralization process employed by LifeNet; and also shown to minimize accompanying protein extraction [10]. The cortical bone particle preparation was suspended in 0.5 M HCl at room temperature for 5 minutes with constant stirring; after which the suspension was centrifuged at 11,000 g for 10 minutes and the S1a supernate was collected. The residual bone particle pellet was again re-suspended in fresh 0.5 M HCL and the procedure repeated to obtain the S1b supernate; followed by repeated extractions to obtain S1c, S1d, and S1e supernates; the total of which collectively comprise the S1 extraction of the mineral component (i.e., the apatite component) of the bone particle preparation. As shown in Figure 1, extractable calcium was successively decreased and was complete by the end of the demineralization procedure. Therefore, “S1 total” reflects the total extractable calcium obtained by this 0.5 M HCl demineralization process.

The P1 pellet represents the residual demineralized bone preparation, which contains the protein/collagen component. We employed the guanidine-HCl procedure [10] to extract the protein component from the P1 pellet (Figure 1). The method extracts essentially all of the bone collagen, which comprises more than 90% of the extracted proteins.

**Figure 2:**
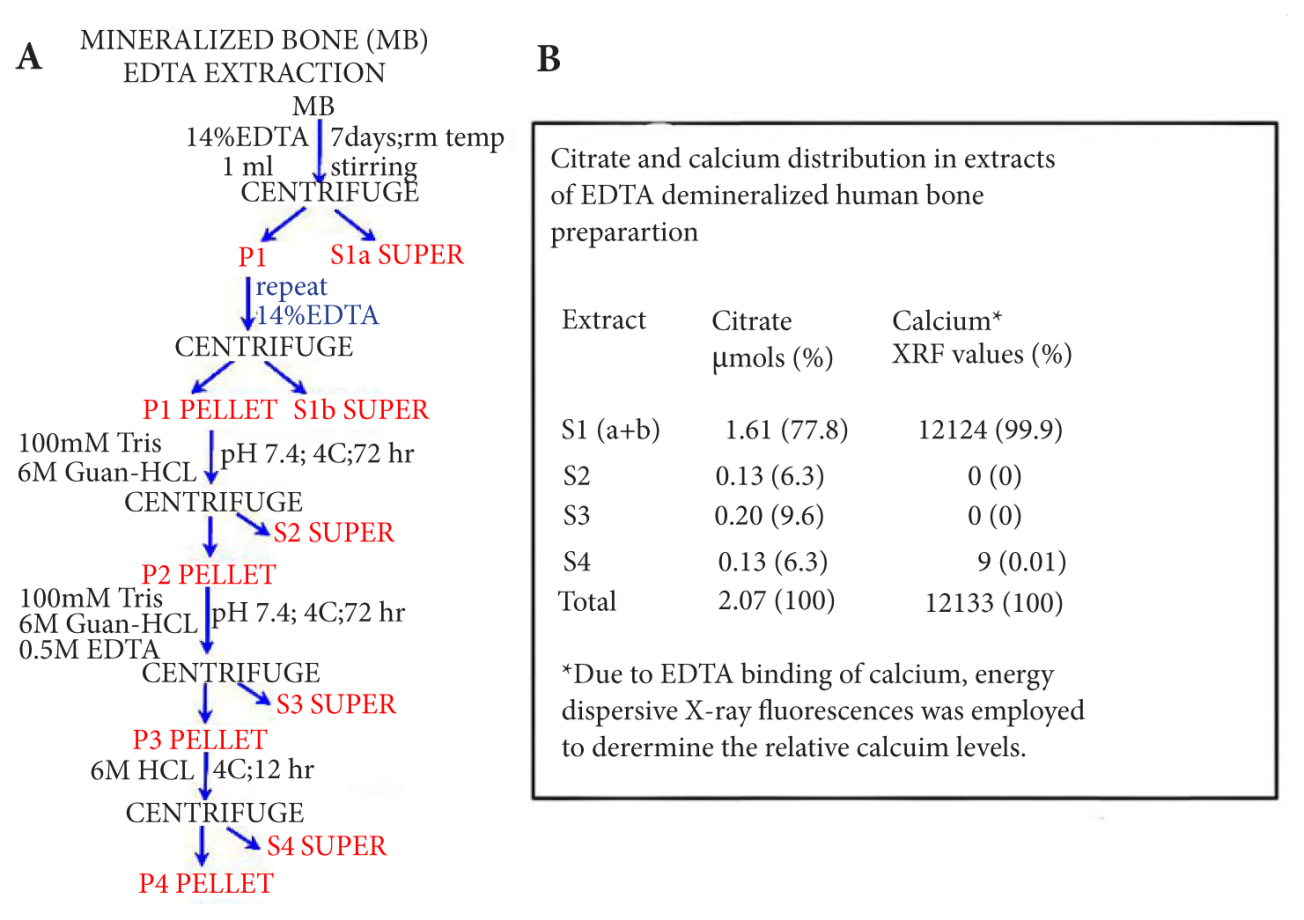
The relative citrate and calcium composition in the mineral and protein extracts of human cortical bone preparation demineralized with EDTA (**A**) The demineralization/protein extraction procedure. (**B**) Citrate and calcium levels of the bone extracts. (% values are % of “Total”).

Figure 1 shows that 90% of the total calcium and 66% of the total citrate are recovered in the mineral extract (S1) of the bone. Nearly all of the remaining citrate is recovered in the guanidine-extracted protein component of bone (S2 and S3); which contained no detectable calcium. While this demonstrates negligible calcium in S2, it does not necessarily reflect the calcium level in S3. We considered that the presence of 0.5 M EDTA in S3 could strongly bind calcium, and therefore would not be detected by the calcium assay chromogen (0-cresolphthalein) that we employed. We determined this possibility by adding known concentrations of calcium to aliquots of the S3 extract, and by adding EDTA to known concentrations of calcium; and observed that EDTA prevented the detection of calcium. Therefore the status of calcium in the S3 extract is unknown; but we deal with this issue in the experiment below (Figure 2).

It was important to establish the expectation that the mineral component contained minimal, if any, extracted collagen; and that the guanidine protein extracts contained virtually all of the collagen component of the bone preparation. The Sirius assay indicated that collagen was negligible in the 0.5 M HCl demineralization S1 extract; and that essentially all of the collagen was extracted by guanidine extracts S2 and S3 (Figure 1). Confirmation was obtained by SDS-PAGE electrophoresis and Western blot analyses of the extracts. The results (Figure 1) demonstrate that the demineralization extract (S1) contained little detectable protein and no detectable collagen. Slight detection of protein is evident in the residual S4 extract; which did not contain detectable collagen; and the guanidine extracts (S2 and S3) contained essentially all of the collagen extracted from the bone. Therefore the demineralization procedure with 0.5 M HCl under the mild conditions achieved the requirement of effective demineralization without accompanying extraction of collagen. Thus, it seems well-founded from these results that the citrate pool (~33% of the total citrate) that resides in the demineralized bone is strongly bound to a collagen complex.

### Citrate composition of cortical bone extracted with EDTA

For further corroboration, we then conducted an experiment in which the HCl extraction of the mineralized cortical bone preparation was replaced by EDTA treatment of the mineralized bone. This procedure eliminates the possible acid hydrolysis and provides a more specific extraction of calcium to relate to citrate extraction. The EDTA extraction (Figure 2) was followed by the same guanidine-HCL extractions of the residual demineralized bone preparation as employed in the preceding experiment (Figure 1). Since EDTA interferes with the calcium assay method that we employ, we determined calcium in these extracts by energy dispersive x-ray fluorescence analysis. The results (Figure 2B) show that essentially all of the extractable calcium in the mineralized bone preparation was recovered in the EDTA S1 extract; along with ~78% of the total citrate. The Sirius assay of S1 (not shown) was negative; thereby demonstrating the absence of extracted detectable collagen by the EDTA demineralization procedure.

The guanidine extracts (S2 and S3) contained ~16% of the total citrate and no detectable calcium. However the residual protein extract S4 exhibited some citrate and calcium; which is possibly due to calcium and citrate release from some collagen following the guanidine extraction. Nevertheless, the EDTA extraction of calcium revealed the existence of two pools of citrate very similar to that obtained by the 0.5N HCl demineralization procedure. Collectively, these results reveal that ~75% of the citrate component in bone is associated with the apatite nanocrystal structure; and ~25% of the total citrate is complexed predominantly with the collagen component of the residual protein in the demineralized bone.

### Citrate composition of LifeNet demineralized cortical bone

The preceding experiments were conducted with the mineralized human cortical bone preparations, which we demineralized in accordance with the procedures described above. In the following experiment, we determined the status of citrate in the demineralized human cortical bone preparation obtained from LifeNet (DGC-Demineralized Cortical Particulate, 250–1000 microns). The demineralized bone particles were subjected to the procedure we employed in Figure 1, which we employed for the mineralized cortical bone preparation. Table 1 shows that the guanidine extracts (S2+S3) contained ~82% of the total citrate and ~98% of the collagen. Thus it is evident that the LifeNet demineralized bone preparation retained the citrate pool that is strongly complexed with collagen; as we identified to exist in mineralized bone. It is also evident that the major pool of citrate that we identified to be incorporated in the apatite component of the mineralized bone has been largely extracted by the demineralization process. The values presented in Table 1 are based on the weight of the demineralized bone preparation, which is not comparable to the preceding experiments based on the weight of mineralized bone preparation. Nevertheless, these observations corroborate the results obtained in the preceding experiments relative to the identification of the pools of citrate.

### Citrate composition of cancellous bone extracted with 0.5 M HCl

The preceding experiments were conducted with human cortical bone preparations. The following experiment was performed with human cancellous bone preparation obtained from LifeNet (OCAN-Mineralized Cancellous Particulate; 250–1,000 micron). The purpose was to determine if the two pools of citrate identified in cortical bone also existed in cancellous bone.

The cancellous bone preparation was extracted as shown in Figure 1A for cortical bone. The results (Table 2) revealed a citrate distribution pattern in cancellous bone that is nearly identical to cortical bone (Figure 1B). The total citrate was comprised of the two major pools identified in cortical bone; i.e., the mineral (apatite) pool, and the tightly bound collagen complex pool. However, whereas the citrate pool in the mineral component of cortical bone represented ~65–80% of the total citrate, in the cancellous bone it comprised ~50% of the total citrate. Therefore, the % of total citrate bound to collagen is greater in cancellous versus cortical bone. Nevertheless, the results with cancellous bone further established the relationship of the presence of a major citrate pool associated with the hydroxyapatite component, and a major citrate pool that is bound to the collagen complex.

## Discussion and conclusion

The presence of high citrate levels in bone has been well established for more than 70 years. Yet, the status of citrate in relation to the chemical/structural organization of bone has remained unknown. In this report, we identify for the first time that the total citrate in mineralized bone consists of two major and distinct pools of citrate. In cortical bone, one pool of citrate is associated with the mineral component of bone and comprises ~65–80% of the total citrate; and another pool of citrate is strongly bound to the collagen complex and comprises 20–35% of the total citrate. However, we must recognize that the former possibly includes some “free” citrate mainly as calcium citrate; which, if so, might comprise ~5–10% of the total citrate as reported to exist in dentine [11]. In any event, this does not detract from the identification of the two major pools of citrate as we have described. Cancellous bone contains similar total citrate as compact bone; and also exhibits the two major pools of citrate although the distribution is of the order of ~50% in each pool. Although this initial study focused on a limited source of human bone preparations, we believe that further studies will demonstrate that this citrate relationship will likely exists in all bones and in all vertebrates.

The identification for the first time of these two major pools of citrate was dependent upon conditions that would permit the demineralization of the bone to obtain the mineral (apatite) component, without extraction of the collagen component as a “contaminant” of the mineral extract. Under such conditions, the residual demineralized bone should retain the citrate pool that is bound to the collagen complex. We achieved these criteria by employing 0.5 M HCl under mild extraction conditions for effective demineralization of the mineralized bone preparation, which did not contain any detectable collagen; followed by extraction of the collagen component of the residual demineralized bone (Figure 1). Similar results were obtained with EDTA extraction under conditions in which the calcium is extracted from the mineralized bone in the absence of accompanying collagen extraction.

HCl is widely used for demineralization; however, higher HCl concentrations, longer extraction periods, and other bone disruptive conditions have generally been employed. Those conditions will result in the extraction of the collagen complex. Notably, the amount of extracted collagen increases greatly when the HCl concentration exceeds 0.5 M [9]. For these reasons, earlier studies (such as [11,12]) failed to identify the pools of citrate as we now describe. Instead, such studies reached conclusions that all or most of the bone citrate existed as a peptide/protein complex; which was extracted with the mineral component. We experienced this when we treated the mineralized bone particles with 1.0 M HCl at 65C followed by homogenization. With this protocol, nearly all (>90%) of the total citrate was extracted with the protein component (including collagen); which is not representative of the in situ status of citrate in bone.

An important issue is the relationship of the two pools of citrate to the structural and functional properties of bone. Some early investigators [13,14] proposed that the citrate in bone is incorporated at the surface of the hydroxyapatite nanocrystal; however, the technology required to establish that relationship did not exist. Hu et al., [4–6] recently reported that the citrate is strongly bound in the apatite nanocrystal at the surface of the collagen fibril; thereby forming an apatite-citrate-collagen nanocomposite. The importance of the citrate incorporation is that it limits the size of the apatite nanocrystal at ~3 nm, which is optimal to obtain the mechanical properties, to increase stability, to prevent fracture; and also to provide better biocompatibility in tissue repair [4–6]. In a subsequent report, Davies et al., [7] proposed that the citrate bridges the layers of mineral platelets that comprise the mineral component of bone. Seemingly, these are opposing views when one assumes that a single pool of citrate exists in the apatite nanocrystal/collagen complex. However, our identification of the two major pools of citrate supports the presence of a citrate pool in the apatite (mineral) component, and another citrate pool that is tightly bound to the apatite nanocrystal/collagen complex.

**Figure 3:**
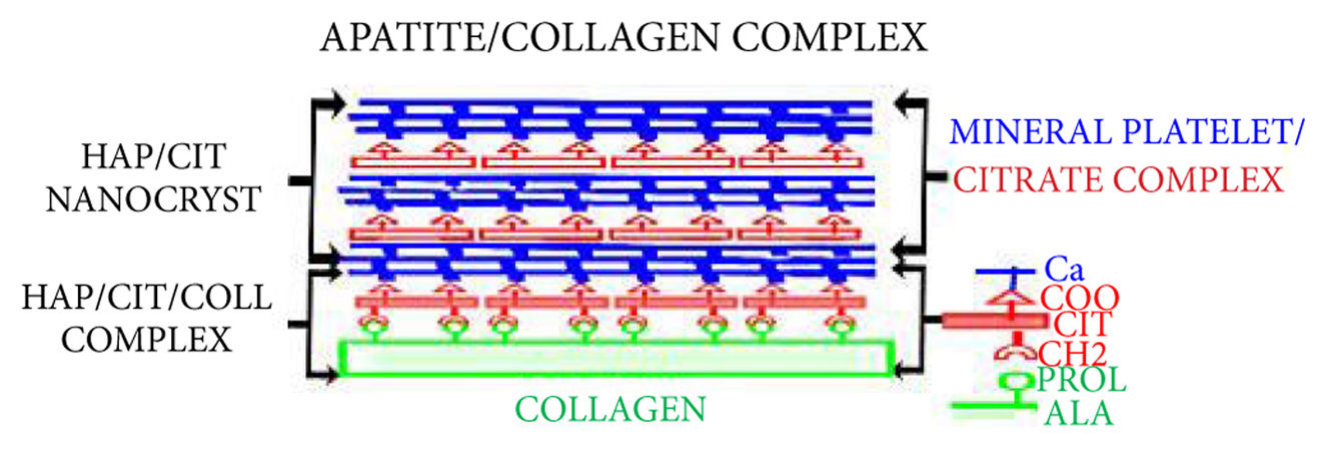
Concept of the incorporation of two pools of citrate in the structure of the apatite nanocrystal/collagen complex.

The integration of our results with the observations and interpretations presented by the studies of Hu et al., [4–6,15] and Davies et al., [7], leads us to propose a new concept as is represented in Figure 3. This concept considers that most of the citrate in cortical bone (and ~50% in cancellous bone) is associated with the mineral component, possibly as proposed by Davies et al., The concept also supports the view of Hu et al., that there exists a citrate component which is strongly bound as a collagen complex. This seems evident from the existing pool of citrate that is not extracted by the demineralization process; and which appears with the extraction of collagen from the resulting demineralized residue. While this concept seems reasonable based on presently available information, we recognize that subsequent research is required to establish its validity, or to require modification of the concept. In any event, it is becoming increasingly evident that the requirement for citrate incorporation in normal bone formation is as essential as calcium or any other recognized component. It is apparent that the importance of citrate incorporation in bone should no longer be ignored or minimized.

**Figure 4:**
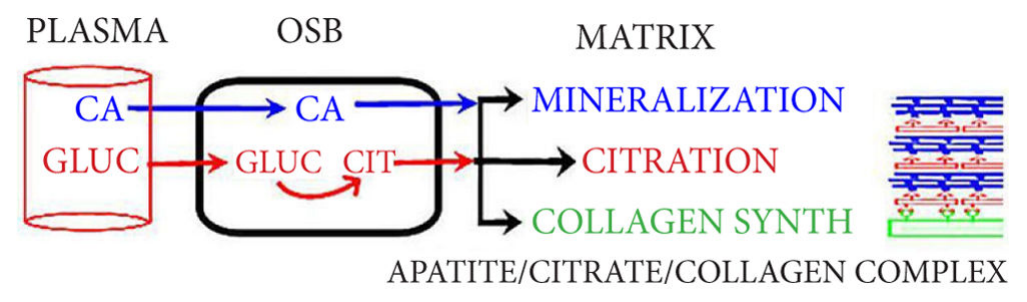
The concept of the role of the osteoblasts for citrate production and the process of citration for the incorporation of citrate in bone formation.

It also becomes evident that the incorporation of citrate in bone is not a random process; but must be coordinated with mineralization during formation of the hydroxyapatite/collagen complex. However, the source of citrate required to achieve this event during bone formation remains unknown. A widely held early and contemporary view is that the blood plasma citrate is the source of citrate in bone; especially in combination with the transport of calcium from plasma into bone. However, evolving evidence casts serious doubt regarding plasma citrate as the source of citrate [2,16]. Instead, the more likely source of citrate is its synthesis and production by the osteoblasts during bone formation. This is supported by our recent studies, which now demonstrate that the osteoblasts are specialized functional metabolic citrate-producing cells; and that this capability occurs during osteogenic differentiation of the mesenchyme cells [2,16]. In addition, the osteoblasts do not exhibit expression of the citrate transporter (NaCT; Slc13A5) that would be necessary for the transport of citrate from plasma [16]. Therefore, osteoblast de novo citrate production and incorporation into bone (“citration”) likely occurs in concert with mineralization during bone formation as represented in Figure 4. This concept of the formation of a mineralization-citration-collagen complex should replace the conventional contemporary view, which has excluded citrate incorporation from the apatite-collagen complex in bone formation.

The concept provides a new understanding that should be considered in relation to the factors and conditions associated with the formation of new bone that represents the structural-functional properties of normal bone. It is also important for understanding the implications of citrate in bone disorders. For example, vitamin D-deficient rickets is characterized by loss of bone citrate, and is treatable by vitamin D and citrate therapy [1,17–20]. Yet, the role of vitamin D in bone citrate metabolism and production remains unknown. Also, it is highly likely that the loss of citrate contributes to the problem of bone fractures associated with osteoporosis; especially since vitamin D and zinc (also involved in citrate metabolism [2]) are implicated in osteoporosis treatment [21,22]. However, no reported studies of citrate in osteoporosis exist.

The results of this study conducted with mineralized and demineralized human bone preparations calls attention to the emerging development of the employment of implanted bone preparations, synthetic platforms, and/or stem cell therapy to induce osteogenesis for the generation of new and replacement bone. An optimal goal of bone regeneration is that the osteoinduced bone product should exhibit the structural/functional/biomechanical properties of the normal native bone. This cannot be achieved if the regenerative bone process does not include the appropriate incorporation of citrate into the structure of the new bone. However, there is no information or reported studies relating to the citrate content and its incorporation in osteoinductive bone products that results from implanted human bone preparations, or synthetic platforms, or stem cell therapy. Also no information exists regarding the conditions and factors that might be required to induce the “citration” process in regenerating bone. In summary, the evaluation of the “quality” of the newly generated bone requires the determination of the status of citrate.

## Figures and Tables

**Table 1:** Citrate, calcium and collagen composition of life net demineralized cortical bone extracts.

Extract	CITRATE μmols (%)	CALCUIM μmols (%)	COLLAGEN* rel values (%)
S1	0.36 (7.2)	5.0 (65.8)	3.0 (0.9)
S2	0.13 (2.6)	2.5 (32.9)	148.2 (44.1)
S3	3.97 (79.4)	0 (0)	180.9 (53.7)
S4	0.54 (10.8)	0.1 (1.3)	4.6 (1.3)
Total	5.00 (100)	7.6 (100%)	336.7 (100)

% is the % of the “Total” values

* Collagen values are relativesirius red assay values.

**Table 2:** Citrate and calcium distribution in human mineralized cancellous bone extracts.

Extract*	Citrate μmols (%)	Calcium μmols (%)
S1a	0.549	99.1
S1b	0.316	38.8
S1c	0.147	17.0
S1d	0.097	0
S1e	0.054	0
S1 total	1.163 (51)	154.9 (100)
S2	0.401 (19)	0 (0)
S3	0.633 (28)	0 (0)
S4	0.029 (1)	0 (0)
Total	2.226 (100)	154.9 (100)

* Extracts are described in Figure 1. %=% of Total.
